# Case Report of an Acute Myocardial Infarction as a Result of Spontaneous Coronary Artery Dissection in a Patient with Fibromuscular Dysplasia

**DOI:** 10.1155/2019/3051616

**Published:** 2019-05-02

**Authors:** A. Kalinskaya, D. Skrypnik, A. Kostin, E. Vasilieva, A. Shpektor

**Affiliations:** Moscow State University of Medicine and Dentistry Named After A.I. Evdokimov, Clinical Hospital Named After I.V. Davidovsky, Moscow, Russia

## Abstract

**Background:**

Spontaneous coronary artery dissection (SCAD) is an underdiagnosed and rare cause of myocardial infarction (MI). SCAD is defined as the separation of the coronary artery wall by hemorrhage with or without intimal tear. It causes acute coronary syndrome in 1.7% to 4% of cases.

**Case Summary:**

We report a case of a patient with acute MI caused by SCAD with marked progression of dissection within 4 days and spontaneous healing in 2 months. Fibromuscular dysplasia (FMD) of the arteries is an associated condition of SCAD that was found in our patient.

**Conclusion:**

In young women admitted to the clinic with signs of acute myocardial infarction, SCAD should be suspected. FMD as an associated condition that should be ruled out in every SCAD patient. Conservative treatment of SCAD is the most preferable strategy.

## 1. Introduction

Spontaneous coronary artery dissection (SCAD) is an underdiagnosed and rare cause of myocardial infarction (MI). SCAD is defined as the separation of the coronary artery wall by hemorrhage with or without intimal tear. SCAD causes acute coronary syndrome in 1.7% to 4% of cases [1]. We report a case of a patient with acute MI caused by SCAD with marked progression of dissection within 4 days and spontaneous healing in 2 months.

## 2. Case Report

A 47-year-old woman with a 4-day history of chest pain, which had begun directly after emotional stress (air travel), was transferred to our clinic from another hospital. The patient was normosthenic, had practiced gymnastics in youth, and had no signs of joint hypermobility syndrome or other connective tissue disorder. She had smoked for a long time and had arterial hypertension grade 2. She had never experienced chest pain before and was very active in her everyday life.

**Figure 1:**
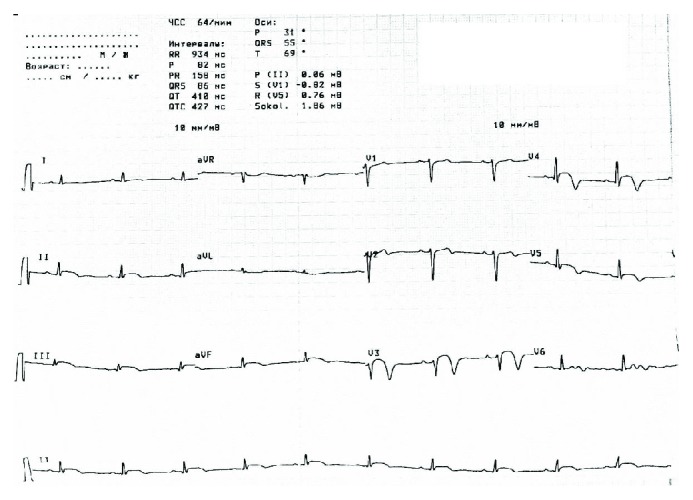
Electrocardiogram revealed a sinus rhythm with a 2 mm ST elevation in leads II, III, aVF, V3-V5, “-” and T V3-V5.

**Figure 2:**
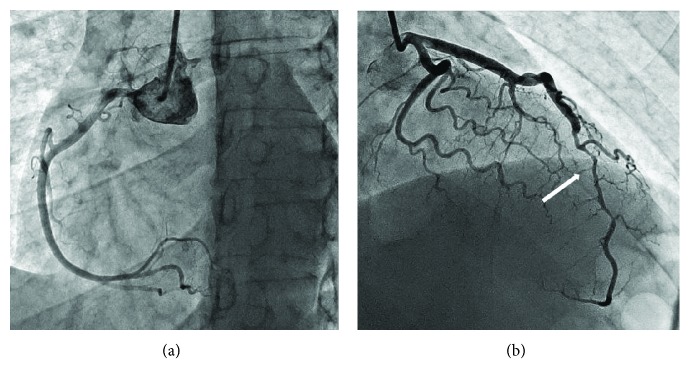
The first coronary angiography revealed a long segment disease of the LAD artery. Right coronary artery is normal.

**Figure 3:**
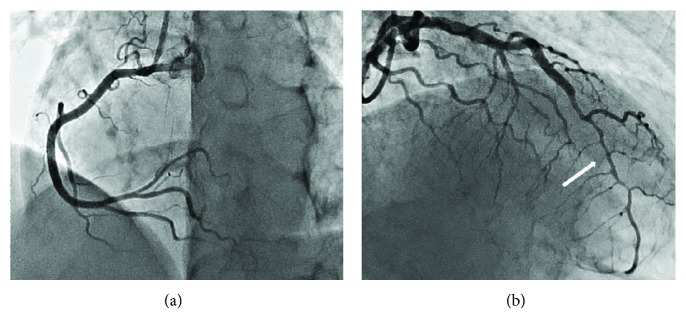
Coronary angiography view 4 days after admission with dissection of the LAD.

An electrocardiogram upon admission to our clinic revealed a sinus rhythm with a 2 mm ST elevation in leads II, III, aVF, V3-V5, “-” and T V3-V5 (Figure 1). On echocardiography, an apex dyskinesis was found; the left ventricle ejection fraction was 56%. The level of cardiac troponin-I was 13.2 ng/mL, confirming the development of acute myocardial infarction. We analysed the first angiogram, which had been performed in another clinic. It revealed 70–75% smooth extended narrowing in the middle segment of the left anterior descending artery, and no signs of atherosclerosis in the proximal segment of LAD or the remaining arteries were found. Side branches were absolutely normal as well (Figure 2). It looked like a subintimal hematoma. We repeated the coronary angiography upon admission on day 4; the extension of the culprit lesion to the distal segment of the LAD was observed (Figure 3). Because of the high risk of intima rupture, intravascular visualization was not performed. This finding on the second angiogram confirmed our initial suspicion and corresponded to SCAD type 2.

**Figure 4:**
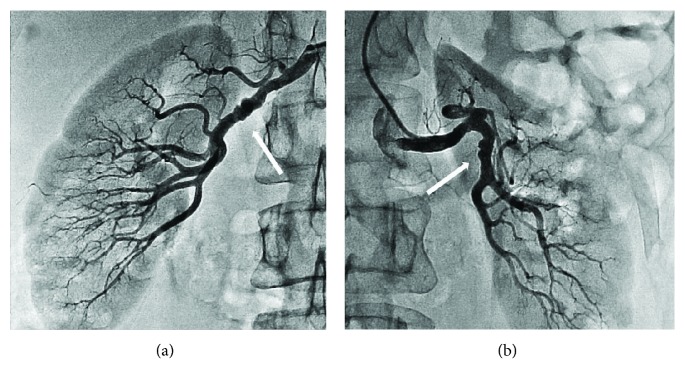
Arteriogram with contrast showing fibromuscular dysplasia of the middle right and left renal arteries.

**Figure 5:**
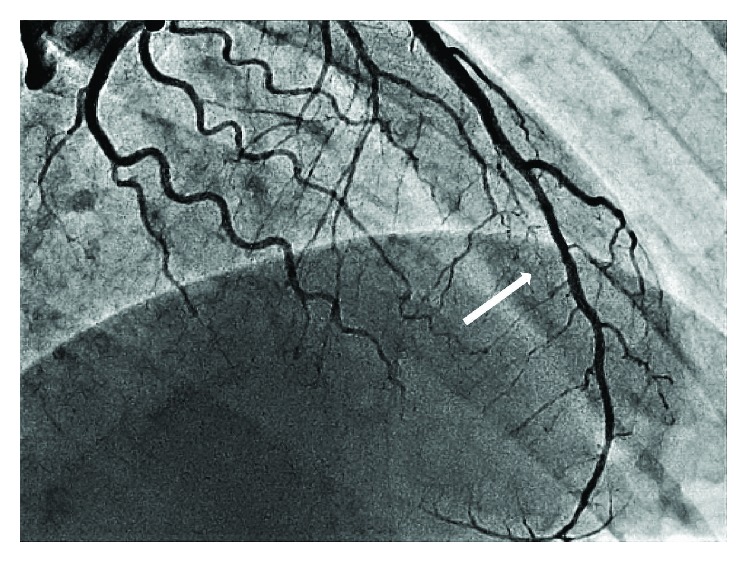
The last coronary angiography shows near-normal left coronary artery.

Considering that the association of SCAD and FMD is a well-known fact, we performed angiography of the renal and carotid arteries [2]. In the mid and distal portions of the renal arteries, alternating dilatation and constriction (string-of-beads) were observed (Figure 4), confirming a diagnosis of fibromuscular dysplasia (FMD). The patient received conservative treatment only (metoprolol 25 mg/day and aspirin 75 mg/day). In two months, a follow-up visit took place. The patient had no episodes of chest pain or dyspnea. Repeated coronary angiography revealed absolutely normal coronary arteries (Figure 5).

## 3. Discussion

The patient developed acute MI without any signs of atherosclerosis. SCAD, defined as the separation of the coronary artery wall by hemorrhage, is a rare underlying cause of acute coronary syndrome [3, 4]. There are three known types of SCAD. Type 1 is characterized by the appearance of a double lumen under contrast staining of the arterial wall. Type 2 appears as diffuse (typically 20 to 30 mm) and smooth narrowing that can vary in severity (due to intramural hematoma). Type 3 appears as focal or tubular stenosis that mimics atherosclerosis and can be confirmed only under intravascular visualization [1, 5].

SCAD is often underdiagnosed and mistaken for atherosclerosis. In order to establish the challenging diagnosis of SCAD, the specific flowchart was developed [6]. This flowchart includes clinical and angiographic features of SCAD. In 2017, Motreff et al. identified the specific five angiographic features of SCAD: (1) absence of atheroma on other coronary arteries; (2) radiolucent flap(s); (3) contrast dye staining of the arterial wall; (4) starting and/or ending of the angiographic ambiguity on a side branch; and (5) long narrowing of the lumen calibre: smooth and linear, or stenosis of varying severity mimicking a “stick insect” or “radish” aspect [7]. In this case, we found the following: (1) typical smooth narrowing of the coronary artery, (2) progress of artery dissection in the distal direction in 4 days, (3) no signs of atherosclerosis in the remaining arteries, and (4) dissection ended on a side branch. All these findings were conclusive for SCAD. This case demonstrates the rapid progression of SCAD leading to acute MI.

The acute and long-term management of SCAD is not well developed, as there have been no randomized trials assessing the optimal strategy. According to observational studies, most SCAD lesions heal spontaneously [8]. For this reason, the preferred strategy in SCAD treatment is conservative [4]. We chose medical treatment as well. According to data in the literature, the full resolution of SCAD occurs in 12 to 40 months [9, 10]. Our patient was doing well after 2 months; that is why we decided to repeat the control angiography well before 12 months. It revealed a healed coronary artery.

SCAD is often accompanied by FMD (in 62–86% of cases) [11]. The recent published study showed that SCAD may have a genetic link to FMD. There is an increased risk for both SCAD and FMD in patients carrying the rs9349379-A allele of PHACTR1/EDN1 genetic locus (chromosome 6q24) [2].

FMD is a nonatherosclerotic vascular disease that results in occlusion, aneurysm, or dissection of the artery. The most commonly affected vascular bed is the renal or carotid arteries (65% of cases) [12]. The exact frequency of FMD of coronary arteries is still unknown, as the diagnosis of coronary FMD is rather challenging. The string-of-beads appearance is rarely seen. In most cases, we can see distal tapering, nonatherosclerotic stenosis, or dissection of the artery [13]. To confirm the diagnosis of coronary FMD, the visualization of another vascular bed is necessary. In our case, we found the typical string-of-beads in renal arteries.

## 4. Conclusion

In young women admitted to the clinic with signs of acute myocardial infarction, SCAD should be suspected. FMD as an associated condition should be ruled out in every SCAD patient. Conservative treatment of SCAD is the most preferable strategy.
